# Which endometrial preparation protocol provides better pregnancy and perinatal outcomes for endometriosis patients in frozen-thawed embryo transfer cycles? A retrospective study on 1413 patients

**DOI:** 10.1186/s13048-023-01095-4

**Published:** 2023-01-09

**Authors:** Yaxin Guo, Zishui Fang, Lin Yu, Xin Sun, Fei Li, Lei Jin

**Affiliations:** 1 Reproductive Medicine Center, Tongji Hospital, Tongji Medical College, Huazhong University of Science and Technology, 1095 JieFang Avenue, Wuhan, 430030 People’s Republic of China; 2 Department of Artificial Intelligence, NanPeng Artificial Intelligence Research Institute Ltd, Chongqing, People’s Republic of China

**Keywords:** Endometrial preparation, Endometriosis, Clinical pregnancy, Perinatal outcome

## Abstract

**Objective:**

To determine the optimal endometrial preparation protocol for a frozen embryo transfer in patients with endometriosis.

**Design:**

Retrospective cohort study.

**Setting:**

Tertiary care academic medical center.

**Patient(s):**

One thousand four hundred thirteen patients with endometriosis who underwent oocyte aspiration from 2015 to 2020 and frozen embryo transfer from 2016 to 2020 and received natural cycle, hormone replacement treatment with or without GnRHa pretreatment endometrial preparation.

**Intervention(s):**

None.

**Main outcome measure(s):**

Clinical pregnancy rate, live birth rate, miscarriage rate, multiple pregnancy rate, biochemical pregnancy rate and ectopic pregnancy rate. Singleton live births were assessed for perinatal outcomes and obstetric complications.

**Result(s):**

There were no differences in clinical pregnancy outcomes or prenatal outcomes among the three commonly used endometrial preparation protocols for frozen embryo transfer cycles in patients with endometriosis. Results remained after screening variables using univariate logistic regression into multivariate logistic regression. No advantages or disadvantages were found among the three endometrial preparation protocols in patients with endometriosis.

**Conclusion(s):**

Natural cycle, hormone replacement cycle, or hormone replacement treatment with GnRHa pretreatment showed no superiority or inferiority in pregnancy and perinatal outcomes in patients with endometriosis.

**Supplementary Information:**

The online version contains supplementary material available at 10.1186/s13048-023-01095-4.

## Introduction

Endometriosis, a common health problem affecting 10% of women of reproductive age, is defined as the presence of endometrium-like tissue outside the uterus [1]. Numerous studies have demonstrated cellular and molecular differences in the eutopic endometrium of patients with and without endometriosis, which may lead to altered endometrial receptivity [2, 3]. Many women whose fertility are impaired by endometriosis require treatment with assisted reproductive technology (ART) to achieve pregnancy outcomes. However, the optimal endometrial preparation protocol in frozen-thawed embryo transfer (FET) cycles for patients with endometriosis is rarely discussed.

Despite the increase in FET, the most optimal priming regimen of the endometrium in the ART general population remains controversial [4], not to mention that there are few studies on endometriosis [5]. In a Cochrane review on the effect of hormonal treatment prior to ART, the authors conclude that administration of gonadotropin-releasing hormone agonists (GnRHa) for a period of 3–6 months in women with endometriosis increases the live birth rate and clinical pregnancy rate [6]. However, the updated version of this review showed uncertain results as to whether long-term GnRHa therapy impacts on the live birth rate or indeed the complication rate [7]. Moreover, one study suggested that long-term pituitary downregulation before frozen embryo transfer could improve pregnancy outcomes in women with adenomyosis [8], which often coexists with endometriosis. Whereas, Muzi Li et al. disagreed [9]. These equivocal results raise the question of whether the endometrial preparation regimen with GnRHa downregulation might also be beneficial for patients with endometriosis. To the best of our knowledge, there are still no clinical studies on this issue.

The present study was conducted to assess the effect of different endometrial preparation regimens on pregnancy and perinatal outcomes in women with endometriosis after FET cycles. In this study, we performed a retrospective cohort study to compare the pregnancy outcomes and perinatal outcomes of singletons conceived after FET with natural cycle (NC), hormone replacement treatment (HRT) with or without GnRHa pretreatment in women with endometriosis.

## Materials and methods

### Patients

This was a retrospective cohort study of patients with endometriosis who underwent oocyte aspiration at the Reproductive Medical Center of Tongji hospital from 2015 to 2020 and underwent FET cycles in our center from 2016 to 2020. Inclusion criteria included a normal uterine cavity as assessed by ultrasonography, hysterosalpingography, or hysteroscopy, and high-quality frozen embryos were transferred. Patients with endometriosis were diagnosed by laparoscopy or laparotomy.

Non-autologous, PGD, and canceled cycles were excluded. In our reproductive medicine center, three endometrial preparation protocols were mainly applied for women with endometriosis namely NC, HRT, and hormone replacement treatment with GnRHa pretreatment (GnRHa + HRT). FET cycles with other endometrial preparation protocols were also excluded (Supplemental Fig. 1). This study was approved by the Institutional Review Board of Tongji Hospital, Tongji Medical College, Huazhong University of Science and Technology (reference: TJ-IRB20211291).

### Endometrial preparation before embryo transfer

Three commonly used endometrial preparation protocols have been described elsewhere [10–12]. From 2016 to 2020, 74 patients with endometriosis underwent natural cycle FET (NC group). Following spontaneous menstruation, endometrial thickness, follicular development, and ovulation were evaluated by transvaginal ultrasound examination (USE) and the serum progesterone levels were measured starting on days 10–12 of the menstrual cycle. FET was planned for 3 days after ovulation, indicated by serum progesterone > 5 ng/mL. Intramuscular administration of progesterone for luteal support was started from 1 day after ovulation. For hormone replacement treatment cycles (HRT group), oral administration of estradiol (Progynova; Bayer Schering Pharma AG, Germany) was initiated with 2 mg/day from cycle days 1 to 4, 4 mg/day from days 5 to 8, and 6 mg/day from days 9 to 12. Similarly, the endometrial thickness and ovulation were assessed from day 13, and the estradiol dose was adjusted based on the endometrial thickness. 40 mg intramuscular administration of progesterone was administered and maintained for the following 3 days when the endometrial thickness reached at least 8 mm. Embryo transfer was conducted on day 4, after 3 days of progesterone administration. For HRT with GnRHa pretreatment cycles (GnRHa + HRT group), GnRHa including triptorelin and leuprorelin was injected at a dose of 3.75 mg on day 2 of menstruation. After a follow-visit 28 days later, patients started their HRT cycles as previously described.

Embryo culture, vitrification and warming were implemented as previously published [11, 13].

### Outcomes

In this study, both pregnancy outcomes and prenatal outcomes were considered. Live birth was defined as the delivery of at least one viable infant. Clinical pregnancy was classified as those cycles resulting in the identification of a gestational sac with fetal heart activity on USE. A positive pregnancy test that did not result in a clinical pregnancy was referred to as a biochemical pregnancy loss. Miscarriage was defined as spontaneous loss after sonographic visualization of an intrauterine gestational sac. The primary outcome was the live birth rate (LBR) and clinical pregnancy rate (CPR).

In order to reduce bias due to vanishing twin pregnancies, only live births from singleton pregnancies were selected for analysis of perinatal outcomes. Low birth weight (LBW) was defined as birth weight of fewer than 2500 g while macrosomia was defined as birth weight of more than 4000 g. Small for gestational age (SGA) was defined as birthweight <10th percentile while large for gestational age (LGA) was defined as birthweight >90th percentile of reference standard birthweight for gestational age, which was based on Chinese populations and adjusted for sex and gestational age [14]. Preterm birth (PTB) was defined as live birth before 37 weeks gestation. Information regarding all abnormal perinatal outcomes was obtained by a follow-up telephone interview and entered into the electronic database.

### Statistical analysis

SAS 9.4 and R 4.1.2 were utilized for data analysis in this study. All continuous variables were assessed for normality of distribution by the Shapiro-Wilk test. The median and interquartile range was used for continuous variables since none of them were normally distributed, and categorical data were expressed as frequency and percentage. Patient characteristics and outcomes were summarized descriptively. Continuous variables were assessed by the Kruskal-Wallis test, and categorical variables were analyzed by using the chi-squared test or Fisher’s exact test, as appropriate. Missing values are imputed with median due to missing at random. To evaluate the association between the different endometrial preparation protocols and pregnancy outcomes and perinatal outcomes, confounding variables should be identified and adjusted. Univariate logistic regressions between each outcome and all covariates were firstly applied to identify covariates that effect the outcomes. Then, multivariate logistic regressions were conducted where statistically significant covariates with significance levels less than 0.2, clinically significant variables and the exposure of interest were considered. In the multivariate logistic regressions for pregnancy outcome, we always included the exposure of interest endometrial preparation protocols, and three clinically significant variables including maternal age at FET, BMI and infertility diagnosis. For prenatal outcomes, maternal age at FET, BMI, and endometrial preparation protocols were fixed in the model. The variable selection was done using backward method. Unadjusted odds ratios (ORs) and adjusted odds ratios (AORs) were reported with their 95% confidence intervals (95% CIs) to demonstrate the level of overall association. Bonferroni correction was used for multiple comparisons. The sensitivity analysis was conducted excluding patients with any missing data to test the accuracy of the analysis. All statistical tests were two-sided, and the level of statistical significance was set at *P* < 0.05.

## Results

### Baseline and cycle characteristics

A total of 1413 patients who fulfilled the inclusion and exclusion criteria were included in this analysis, which were grouped according to the endometrial preparation protocols. There were 389, 950, and 74 patients in GnRHa + HRT group, HRT group and NC group, respectively. The baseline characteristics of the patient cohort are detailed in Table 1, which revealed statistically significant differences (*p* < 0.05) for AMH, AFC, infertility duration, and infertility etiology.

**Table 1:** General characteristics and clinical outcomes of patients with different endometrial preparation protocols

Variable	GnRHa + HRT (*N* = 389)	HRT (*N* = 950)	NC (*N* = 74)	*P* value
Maternal age at oocyte retrieval, y	31.0 (29.0, 35.0)	31.0 (29.0, 35.0)	32.0 (30.0, 35.0)	0.201^3^
Body mass index, kg/m^2^	20.8 (19.4, 22.5)	20.8 (19.5, 22.8)	21.1 (19.6, 22.6)	0.742^3^
Baseline FSH, mIU/mL	7.8 (6.6, 9.6)	7.8 (6.5, 9.0)	7.9 (6.4, 9.8)	0.665^3^
Antral follicle count (AFC)	8.0 (5.0, 11.0)	9.0 (6.0, 14.0)	7.0 (4.0, 13.0)	<.001^3*^
AMH level, ng/ml	2.4 (1.4, 4.5)	2.8 (1.7, 5.3)	2.1 (1.2, 4.0)	<.001^3*^
Duration of infertility, years	2.0 (1.5, 4.0)	3.0 (2.0, 4.0)	3.0 (2.0, 5.0)	0.006^3*^
Infertility diagnosis				0.969^1^
Primary infertility, n (%)	257 (66.1%)	629 (66.2%)	50 (67.6%)	
Secondary infertility, n (%)	132 (33.9%)	321 (33.8%)	24 (32.4%)	
Infertility etiology, n (%)				
Male factor	64 (16.5%)	167 (17.6%)	19 (25.7%)	0.161^1^
Female factors				
Tubal factor	189 (48.6%)	472 (49.7%)	35 (47.3%)	0.881^1^
Ovulatory	3 (0.8%)	42 (4.4%)	1 (1.4%)	0.001^2*^
Diminished ovarian reserve	115 (29.6%)	218 (22.9%)	19 (25.7%)	0.039^1*^
Uterine malformation	105 (27.0%)	187 (19.7%)	21 (28.4%)	0.006^1*^
Unexplained/Other	0 (0.0%)	6 (0.6%)	1 (1.4%)	0.115^2^
Ovarian stimulation protocols, n (%)				<.001^1*^
Long GnRH-a	43 (11.1%)	165 (17.4%)	12 (16.2%)	
GnRH-a ultra-long	98 (25.2%)	342 (36.0%)	25 (33.8%)	
GnRH antagonist	146 (37.5%)	198 (20.8%)	16 (21.6%)	
Other protocols	102 (26.2%)	245 (25.8%)	21 (28.4%)	
Duration of stimulation, days	10.0 (9.0, 11.0)	10.0 (9.0, 11.0)	10.0 (9.0, 11.0)	0.018^3*^
Gonadotropin dose, IU	2625.0 (2100.0, 3150.0)	2475.0 (1912.5, 3000.0)	2625.0 (2137.5, 3150.0)	0.011^3*^
No. of oocytes retrieved	9.0 (5.0, 14.0)	10.0 (6.0, 16.0)	8.0 (5.0, 14.0)	0.003^3*^
No. of MII oocytes	7.0 (4.0, 12.0)	9.0 (5.0, 14.0)	7.0 (4.0, 12.0)	0.001^3*^
Oocyte maturation rate	0.9 (0.8, 1.0)	0.9 (0.8, 1.0)	0.9 (0.8, 1.0)	0.478^3^
Fertilization, n (%)				0.070^1^
IVF	105 (27.0%)	199 (20.9%)	23 (31.1%)	
ICSI	266 (68.4%)	698 (73.5%)	48 (64.9%)	
Rescue ICSI	18 (4.6%)	53 (5.6%)	3 (4.1%)	
The number of 2PN	5.0 (3.0, 9.0)	6.0 (4.0, 10.0)	5.0 (3.0, 10.0)	0.012^3*^
Normal fertilization rate	0.7 (0.5, 0.9)	0.7 (0.5, 0.8)	0.7 (0.5, 0.9)	0.187^3^
Blastocyst formation rate	0.8 (0.5, 1.0)	0.7 (0.5, 0.9)	0.7 (0.5, 1.0)	0.025^3*^
Maternal age at FET, y	32.0 (29.0, 35.0)	32.0 (29.0, 35.0)	33.0 (31.0, 35.0)	0.063^3^
Interval between FET and IVF/ICSI, days	125.0 (85.0, 248.0)	91.0 (60.0, 172.0)	112.0 (63.0, 263.0)	<.001^3*^
No. of embryos thawed				0.226^2^
1	242 (62.2%)	537 (56.5%)	44 (59.5%)	
2	143 (36.8%)	393 (41.4%)	28 (37.8%)	
> = 3	4 (1.0%)	20 (2.1%)	2 (2.7%)	
No. of surviving embryos				0.194^2^
1	243 (62.5%)	540 (56.8%)	44 (59.5%)	
2	143 (36.8%)	394 (41.5%)	28 (37.8%)	
3	3 (0.8%)	16 (1.7%)	2 (2.7%)	
total no. of surviving embryos/no. of embryos thawed	1.0 (1.0, 1.0)	1.0 (1.0, 1.0)	1.0 (1.0, 1.0)	0.528^3^
No. of embryos transferred, n (%)				0.324^1^
1	249 (64.0%)	568 (59.8%)	47 (63.5%)	
2	140 (36.0%)	382 (40.2%)	27 (36.5%)	
Type of embryo transferred, n (%)				0.029^1*^
Cleavage embryo	142 (36.5%)	296 (31.2%)	32 (43.2%)	
Blastocyst	247 (63.5%)	654 (68.8%)	42 (56.8%)	
Endometrial thickness, mm	9.7 (8.8, 11.0)	9.2 (8.4, 10.1)	9.7 (8.8, 10.5)	<.001^3*^
Luteal phase support, n (%)				<.001^1*^
Intramuscular injection and oral administration	11 (2.8%)	179 (18.8%)	10 (13.5%)	
Vaginal gel administration and oral administration	179 (46.0%)	387 (40.7%)	26 (35.1%)	
Vaginal suppository administration and oral administration	197 (50.6%)	381 (40.1%)	24 (32.4%)	
Others	2 (0.5%)	3 (0.3%)	14 (18.9%)	
Live Birth, n (%)	156 (40.1%)	384 (40.4%)	30 (40.5%)	0.994^1^
Clinical Pregnancy, n (%)	200 (51.4%)	475 (50.0%)	34 (45.9%)	0.677^1^
Miscarriage, n (%)	44 (11.3%)	91 (9.6%)	4 (5.4%)	0.281^2^
Multiple pregnancy, n (%)	37 (9.5%)	108 (11.4%)	4 (5.4%)	0.221^2^
Biochemical Pregnancy, n (%)	29 (7.5%)	61 (6.4%)	2 (2.7%)	0.321^2^
Ectopic pregnancy, n (%)	2 (0.5%)	7 (0.7%)	0 (0.0%)	>.999^2^

^1^Chi-Square *p*-value; ^2^Fisher Exact *p*-value; ^3^Kruskal-Wallis *p*-value; ^*^P < .05

As for cycle characteristics, ovarian stimulation protocols, gonadotropin dose and duration, the number of retrieved oocytes, MII oocytes and 2PN, blastocyst formation rate, the interval between FET and IVF/ICSI, type of embryo transferred, endometrial thickness and luteal phase support were significantly different in the study groups (*p* < 0.05) (Table 1).

### Clinical pregnancy outcomes

The clinical pregnancy outcomes of the total study population are shown in Table 1. There were no statistically significant differences in any pregnancy outcomes including live birth rate (LBR), clinical pregnancy rate (CPR), miscarriage rate (MR), multiple pregnancy rate (MPR), biochemical pregnancy rate (BPR), and ectopic pregnancy rate (EPR). After univariate analysis (Table 2), predictors with significance levels less than 0.2, endometrial preparation protocols as well as maternal age at FET, BMI and infertility diagnosis were selected for the following multivariate logistic regression. After adjusting for possible confounding factors, no association was found between endometrial preparation protocols and clinical pregnancy outcomes. Sensitivity analysis by excluding patients with any missing data showed similar significance (data not shown).

**Table 2:** Crude and adjusted odds ratios of clinical outcomes

Variable	HRT vs. NC		GnRHa + HRT vs. NC		GnRHa + HRT vs. HRT	
	Crude OR (95% CI)	Adjusted OR (95% CI)	Crude OR (95% CI)	Adjusted OR (95% CI)	Crude OR (95% CI)	Adjusted OR (95% CI)
Live Birth ^a^	1.00 (0.61-1.61)	0.83 (0.49-1.38)	0.98 (0.59-1.63)	0.86 (0.50-1.48)	0.99 (0.78-1.26)	1.04 (0.80-1.35)
Clinical Pregnancy ^b^	1.18 (0.73-1.89)	0.99 (0.60-1.64)	1.24 (0.76-2.05)	1.13 (0.67-1.93)	1.06 (0.84-1.34)	1.15 (0.89-1.48)
Miscarriage ^c^	1.85 (0.66-5.20)	1.80 (0.64-5.07)	2.23 (0.78-6.41)	2.22 (0.77-6.39)	1.20 (0.82-1.76)	1.23 (0.84-1.80)
Multiple pregnancy ^d^	2.24 (0.80-6.27)	1.91 (0.56-6.53)	1.84 (0.64-5.32)	2.24 (0.63-7.92)	0.82 (0.55-1.21)	1.17 (0.70-1.96)
Biochemical Pregnancy ^e^	2.47 (0.59-10.30)	2.31 (0.55-9.66)	2.90 (0.68-12.41)	2.80 (0.65-12.02)	1.17 (0.74-1.86)	1.21 (0.76-1.92)

Odds ratios (ORs) and 95% confidence intervals (CIs) are based on the univariate analysis while adjusted odds ratios (AORs) and 95% CIs are based on the multiple logistic regression model

^a^ Adjusted for maternal age at FET, BMI, infertility diagnosis, diminished ovarian reserve, No. of surviving embryos, type of embryos transferred and endometrial thickness

^b^ Adjusted for maternal age at FET, BMI, infertility diagnosis, diminished ovarian reserve,endometrial thickness, No and type of embryos transferred

^c^ Adjusted for maternal age at FET, BMI, infertility diagnosis, type of embryos transferred

^d^ Adjusted for maternal age at FET, BMI, infertility diagnosis, type of embryos transferred

^e^ Adjusted for maternal age at FET, BMI, infertility diagnosis, type of embryos transferred

### Singleton perinatal outcomes

A total of 465 singleton live births conceived through GnRHa + HRT cycles (*n* = 131), HRT cycles (*n* = 304), and NC (n = 30) were evaluated for obstetric complications and adverse birth outcomes. Patient demographics, treatment factors, and singleton perinatal outcomes are shown in Table 3. The median birth weights for GnRHa + HRT, HRT, and NC ETs were 3.3 kg, 3.4 kg, and 3.2 kg, respectively. The median gestational ages were 38.9 weeks, 39.0 weeks, and 38.6 weeks, respectively. There were no significant differences between the three groups in gestational age, birth weight, delivery mode, gender, abnormal perinatal outcomes, and obstetric complications. After univariate regression analysis and multivariate regression analysis, the results were still retained, with no statistical difference found (Table 4). Sensitivity analysis was conducted by excluding patients with any missing data and showed similar findings (data not shown).

**Table 3:** General characteristics and perinatal outcomes of patients with singleton live births

Variable	GnRHa + HRT (*N* = 131)	HRT (*N* = 304)	NC (*N* = 30)	*P* value
Maternal age at oocyte retrieval, y	30.0 (28.0, 33.0)	30.0 (28.0, 33.0)	32.0 (29.0, 33.0)	0.305^3^
Body mass index, kg/m^2^	20.7 (19.2, 22.1)	20.8 (19.5, 22.6)	21.6 (19.7, 22.9)	0.340^3^
Baseline FSH, mIU/mL	7.7 (6.5, 8.9)	7.5 (6.3, 8.8)	7.7 (6.2, 8.5)	0.757^3^
Antral follicle count (AFC)	8.0 (5.0, 12.0)	11.0 (7.0, 15.5)	9.5 (6.0, 18.0)	<.001^3*^
AMH level, ng/ml	2.7 (1.4, 4.9)	3.3 (2.1, 5.6)	3.0 (1.6, 4.8)	0.014^3*^
Duration of infertility, years	2.0 (1.5, 3.0)	3.0 (1.5, 4.0)	3.0 (2.0, 3.0)	0.284^3^
Infertility diagnosis				0.747^1^
Primary infertility, n (%)	91 (69.5%)	219 (72.0%)	20 (66.7%)	
Secondary infertility, n (%)	40 (30.5%)	85 (28.0%)	10 (33.3%)	
Infertility etiology, n (%)				
Male factor	21 (16.0%)	50 (16.4%)	5 (16.7%)	>.999^2^
Female factors				
Tubal factor	67 (51.1%)	155 (51.0%)	15 (50.0%)	0.994^1^
Ovulatory	1 (0.8%)	12 (3.9%)	1 (3.3%)	0.164^2^
Diminished ovarian reserve	29 (22.1%)	43 (14.1%)	3 (10.0%)	0.082^2^
Uterine malformation	30 (22.9%)	44 (14.5%)	9 (30.0%)	0.022^1*^
Unexplained/Other	0 (0.0%)	3 (1.0%)	0 (0.0%)	0.638^2^
Ovarian stimulation protocols, n (%)				0.005^1*^
Long GnRH-a	21 (16.0%)	66 (21.7%)	8 (26.7%)	
GnRH-a ultra-long	34 (26.0%)	124 (40.8%)	13 (43.3%)	
GnRH antagonist	47 (35.9%)	65 (21.4%)	5 (16.7%)	
Other protocols	29 (22.1%)	49 (16.1%)	4 (13.3%)	
Duration of stimulation, days	10.0 (9.0, 11.0)	10.0 (9.0, 11.0)	10.0 (10.0, 12.0)	0.020^3*^
Gonadotropin dose, IU	2625.0 (2025.0, 3165.0)	2400.0 (1875.0, 3000.0)	2550.0 (2100.0, 3075.0)	0.161^3^
No. of oocytes retrieved	9.0 (5.0, 14.0)	12.0 (8.0, 17.0)	11.0 (7.0, 16.0)	<.001^3*^
No. of MII oocytes	8.0 (4.0, 12.0)	11.0 (7.0, 15.0)	10.0 (6.0, 14.0)	<.001^3*^
Oocyte maturation rate	0.9 (0.8, 1.0)	0.9 (0.8, 1.0)	0.9 (0.9, 1.0)	0.745^3^
Fertilization, n (%)				0.343^2^
IVF	35 (26.7%)	62 (20.4%)	5 (16.7%)	
ICSI	88 (67.2%)	225 (74.0%)	25 (83.3%)	
Rescue ICSI	8 (6.1%)	17 (5.6%)	0 (0.0%)	
The number of 2PN	6.0 (3.0, 8.0)	7.0 (4.5, 10.5)	8.0 (4.0, 11.0)	0.004^3*^
Normal fertilization rate	0.7 (0.6, 0.9)	0.7 (0.6, 0.8)	0.7 (0.6, 0.9)	0.146^3^
Blastocyst formation rate	0.8 (0.6, 1.0)	0.8 (0.5, 0.9)	0.7 (0.5, 0.8)	0.117^3^
Maternal age at FET, y	31.0 (28.0, 34.0)	31.0 (29.0, 33.0)	32.0 (30.0, 33.0)	0.327^3^
Interval between FET and IVF/ICSI, days	131.0 (85.0, 229.0)	90.5 (60.0, 166.0)	80.5 (59.0, 220.0)	<.001^3*^
No. of embryos thawed				0.480^2^
1	19 (63.3%)	187 (61.5%)	89 (67.9%)	
2	10 (33.3%)	113 (37.2%)	41 (32.1%)	
> = 3	1 (3.3%)	4 (1.3%)	1 (0.8%)	
No. of surviving embryos				0.314^2^
1	19 (63.3%)	188 (61.8%)	89 (67.9%)	
2	10 (33.3%)	113 (37.2%)	42 (32.1%)	
> = 3	1 (3.3%)	3 (1.0%)	0 (0.0%)	
total no. of surviving embryos/no. of embryos thawed	1.0 (1.0, 1.0)	1.0 (1.0, 1.0)	1.0 (1.0, 1.0)	0.895^3^
No. of embryos transferred, n (%)				0.816^1^
1	89 (67.9%)	198 (65.1%)	19 (63.3%)	
2	42 (32.1%)	106 (34.9%)	11 (36.7%)	
Type of embryo transferred, n (%)				0.051^1^
Cleavage embryo	35 (26.7%)	52 (17.1%)	8 (26.7%)	
Blastocyst	96 (73.3%)	252 (82.9%)	22 (73.3%)	
Endometrial thickness, mm	10.1 (8.9, 11.3)	9.3 (8.6, 10.3)	9.3 (8.7, 10.4)	<.001^3*^
Luteal phase support, n (%)				<.001^1*^
Intramuscular injection and oral administration	1 (0.8%)	60 (19.7%)	5 (16.7%)	
Vaginal gel administration and oral administration	64 (48.9%)	128 (42.1%)	9 (30.0%)	
Vaginal suppository administration and oral administration	66 (50.4%)	115 (37.8%)	9 (30.0%)	
Others	0 (0.0%)	1 (0.3%)	7 (23.3%)	
Gestational age, wk	38.9 (38.0, 39.4)	39.0 (38.0, 39.6)	38.6 (37.0, 39.0)	0.082^3^
Birth weight, kg	3.3 (3.0, 3.6)	3.4 (3.1, 3.7)	3.2 (2.7, 3.6)	0.237^3^
Delivery mode, n (%)				0.848^1^
Cesarean delivery	114 (87.0%)	260 (85.5%)	25 (83.3%)	
Natural labor	17 (13.0%)	44 (14.5%)	5 (16.7%)	
Gender, n (%)				0.160^1^
Male	81 (61.8%)	159 (52.3%)	15 (50.0%)	
Female	50 (38.2%)	145 (47.7%)	15 (50.0%)	
Low birth weight < 2500 g	9 (6.9%)	19 (6.3%)	2 (6.7%)	0.952^2^
Macrosomia > 4000 g	5 (3.8%)	18 (5.9%)	1 (3.3%)	0.726^2^
Small for gestational age, n (%)	10 (7.6%)	17 (5.6%)	4 (13.3%)	0.194^2^
Large for gestational age, n (%)	21 (16.0%)	51 (16.8%)	5 (16.7%)	0.979^1^
Preterm birth < 37 wk., n (%)	16 (12.2%)	37 (12.2%)	4 (13.3%)	0.945^2^
Gestational diabetes mellitus, n (%)	4 (3.1%)	18 (5.9%)	3 (10.0%)	0.185^2^
Hypertensive disorders of pregnancy, n (%)	125 (95.4%)	291 (95.7%)	29 (96.7%)	>.999^2^
Placenta previa, n (%)	8 (6.1%)	19 (6.3%)	2 (6.7%)	>.999^2^
Fetal malformation, n (%)	2 (1.5%)	11 (3.6%)	1 (3.3%)	0.510^2^

^1^Chi-Square *p*-value; ^2^Fisher Exact *p*-value; ^3^Kruskal-Wallis *p*-value; ^*^P < .05

**Table 4:** Singleton perinatal outcomes of patients in different endometrial preparation protocols

Variable	HRT vs. NC		GnRHa + HRT vs. NC		GnRHa + HRT vs. HRT	
	Crude OR (95% CI)	Adjusted OR (95% CI)	Crude OR (95% CI)	Adjusted OR (95% CI)	Crude OR (95% CI)	Adjusted OR (95% CI)
Delivery mode	1.18 (0.43-3.25)	1.30 (0.47-3.61)	1.34 (0.45-3.98)	1.50 (0.50-4.49)	1.13 (0.62-2.07)	1.15 (0.63-2.11)
Low birth weight < 2500 g^a^	0.93 (0.21-4.22)	2.12 (0.38-11.95)	1.03 (0.21-5.05)	1.45 (0.24-8.63)	1.11 (0.49-2.51)	0.68 (0.28-1.68)
Macrosomia > 4000 g^b^	1.83 (0.24-14.17)	1.74 (0.22-13.77)	1.15 (0.13-10.23)	1.14 (0.13-10.33)	0.63 (0.23-1.74)	0.66 (0.24-1.82)
Small for gestational age^c^, n (%)	0.39 (0.12-1.23)	0.42 (0.13-1.37)	0.54 (0.16-1.85)	0.48 (0.14-1.69)	1.39 (0.62-3.12)	1.14 (0.50-2.62)
Large for gestational age^d^, n (%)	1.01 (0.37-2.77)	0.97 (0.35-2.69)	0.95 (0.33-2.78)	1.10 (0.37-3.26)	0.94 (0.54-1.64)	1.13 (0.64-2.01)
Preterm birth < 37 wk^e^, n (%)	0.90 (0.30-2.73)	0.68 (0.22-2.16)	0.90 (0.28-2.93)	0.68 (0.20-2.31)	1.00 (0.54-1.88)	0.99 (0.51-1.92)
Gestational diabetes mellitus^f^, n (%)	0.57 (0.16-2.05)	0.67 (0.18-2.51)	0.28 (0.06-1.34)	0.27 (0.05-1.31)	0.50 (0.17-1.51)	0.40 (0.13-1.23)
Hypertensive disorders of pregnancy^g^, n (%)	1.30 (0.16-10.25)	1.47 (0.17-12.78)	1.39 (0.16-12.00)	1.65 (0.17-15.87)	1.07 (0.40-2.89)	1.12 (0.39-3.22)
Fetal malformation^h^, n (%)	1.09 (0.14-8.74)	0.96 (0.12-7.88)	0.45 (0.04-5.13)	0.41 (0.04-4.78)	0.41 (0.09-1.89)	0.43 (0.09-1.97)

Odds ratios (ORs) and 95% confidence intervals (CIs) are based on the univariate analysis while adjusted odds ratios (AORs) and 95% CIs are based on the multiple logistic regression model

^a^ Adjusted for maternal age at FET, BMI, AMH, duration of infertility, No. of oocytes retrieved, No. of MII oocytes, endometrial thickness，No. of embryos transferred

^b^ Adjusted for maternal age at FET, BMI, interval between FET and IVF/ICSI

^c^ Adjusted for maternal age at FET, BMI, No. of MII oocytes

^d^ Adjusted for maternal age at FET, BMI, AFC and unexplained/other ovarian stimulation protocols

^e^ Adjusted for maternal age at FET, BMI, ovarian stimulation protocols, normal fertilization rate, and type of embryo transferred

^f^ Adjusted for maternal age at FET, BMI, No. of MII oocytes

^g^ Adjusted for maternal age at FET, BMI,diminished ovarian reserve, normal fertilization rate, and endometrial thickness

^h^ Adjusted for maternal age at FET, BMI and FSH

## Discussion

To the best of our knowledge, this is the first study comparing the impact of different endometrial preparation regimens among patients with endometriosis. According to our results, HRT with GnRHa pretreatment did not show any extra benefit in women with endometriosis compared with HRT and NC, neither in clinical pregnancy outcomes nor perinatal outcomes.

Extensive clinical and molecular data support the existence of biological differences in the eutopic endometrium of women with endometriosis [15–18].The transcriptomic analysis confirmed these earlier observations [19]. Previous studies have reported endometrial gene expression changes associated with defective endometrial receptivity, which reflected a shift away from normal progesterone action and toward excessive estrogen activity [2, 20]. Continuous hypophyseal exposure to GnRHa can deprive the main growth stimulus, therefore long-term therapy with GnRHa has been reported to be effective in treating symptomatic endometriosis [21]. The treatment desensitizes the pituitary gland and contributes to the hypogonadotropic-hypogonadal state, which leads to prolonged amenorrhoea and a low estradiol level. Not only does this improve endometriosis symptoms, but it also reverses the negative effects of endometriosis on ART including poor folliculogenesis leading to decreased oocyte quality, hostile peritoneal environment, and et al. [7]. Various clinical studies have claimed that poor oocyte quality results in impaired implantation rates [22–25].

However, whether this treatment improves fecundity is equivocal. Several studies have investigated the effect of long-term treatment with GnRHa before IVF cycles on women with endometriosis-related infertility [26–29]. In two Cochrane reviews, the authors reach opposite conclusions [6, 7]. Sallam et al. [6] provided evidence of the association of long-term pituitary down-regulation with GnRHa prior to standard IVF/ICSI with higher LBR and CPR in women with endometriosis, making it the first-choice treatment for such patients [30]. On contrary, Georgiou et al. concluded that there was no such benefit and that this treatment was not associated with complication rate and the number of oocytes retrieved, and the number of embryos [7]. In fact, many clinicians are also skeptical of its effectiveness. Given the possible benefit of GnRHa administration for endometriosis and adenomyoma, there are studies focused on different protocols for endometrial preparation during FET cycles. Studies exploring whether GnRHa+ HRT regimen is superior to HRT regimen in patients with adenomyoma have come to different conclusions [8, 9].

In the present study, we analyzed the three most commonly used protocols for endometrial preparation prior to FET in patients with endometriosis. In view of the possible effects of ART on neonatal health [31, 32], prenatal outcomes were also taken into account in addition to pregnancy outcomes. The results showedthat no protocol was superior to the others. Pretreatment of GnRHa did not increase live birth rate and clinical pregnancy rate, but neither did miscarriage rate nor perinatal complications. One possible explanation may be that the GnRHa treatment in our study was only 1 month, whereas previous studies that found benefits tended to treat patients for 3-6 months. Based on the assumption that GnRHa pretreatment would be beneficial to ART in women with endometriosis, 1 month of duration may not be sufficient. What we cannot ignore, however, is that its effect is yet to be explored, so this result is expected. As for the comparison of natural and hormone replacement cycles, varieties of studies have given insights into different patient populations [33–37]. The present study firstly concentrated on endometriosis patients and found no superiority of either protocol.

The study is strengthened by the large cohort size and generally complete baseline and cycle data. Anyway, we cannot exclude a selection bias due to the retrospective design of the study. Although we tried to eliminate confounding factors, it is indisputable that there are inevitably some confounding factors that have not been taken into account. Further randomized control trials are required to determine the impact of the different endometrial preparation protocols in endometriosis patients. Moreover, the importance of investigating the neonatal outcomes and long-term follow-up of children born from frozen embryos [32, 38] and the psychological effects cannot be ignored [39–41]. Notably, there are other overlooked conditions that require the exploration of FET strategies other than endometriosis, such as the diagnosis of cancer [42–45] and thyroid autoimmunity [46–48]. Fertility preservation is a crucial issue to be addressed in all cancer patients of reproductive age, and the safety of different ART strategies for them warrants careful investigation. Furthermore, the role of inositols supplementation in ART has also been reported recently [49–53]. And the influence of cryptic sperm defects on pregnancy outcomes should not be ignored [54].

In conclusion, our findings indicated that endometrial preparation regimen selection of natural cycle, hormone replacement cycle, or hormone replacement treatment with GnRHa pretreatment had no beneficial or detrimental effects on pregnancy and perinatal outcomes in patients with endometriosis.

## Supplementary Information

**Additional file 1: Supplemental Figure 1.** Data selection process.

## Data Availability

The data underlying this article will be shared on reasonable request to the corresponding author.
