# Supplementary figures and images for: Which endometrial preparation protocol provides better pregnancy and perinatal outcomes for endometriosis patients in frozen-thawed embryo transfer cycles? A retrospective study on 1413 patients
Source: J Ovarian Res. 2023 Jan 9;16:7. doi: 10.1186/s13048-023-01095-4 (PMC9830850; doi:10.1186/s13048-023-01095-4)

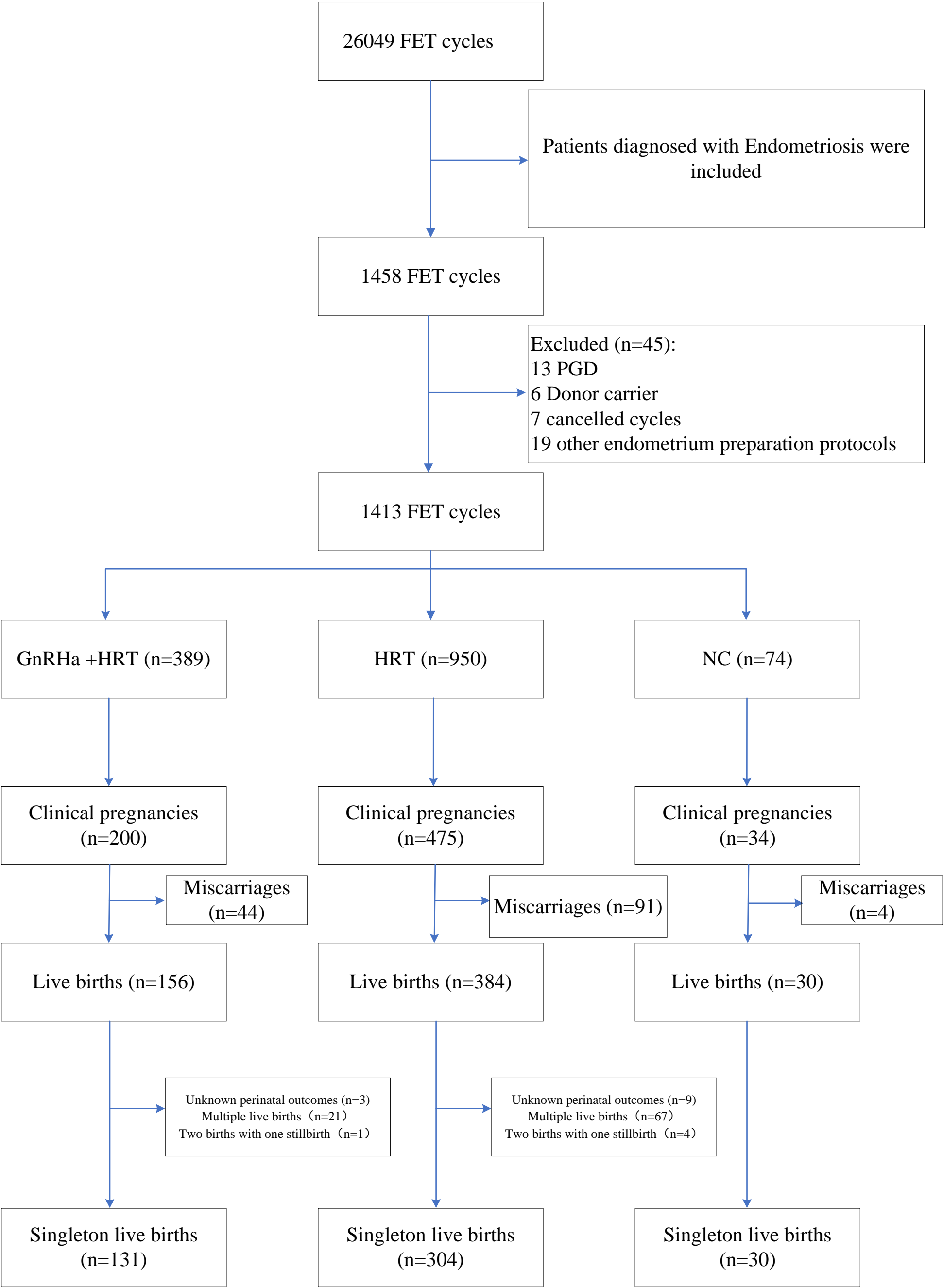

Supplement: Supplementary file 1 — Additional file 1: Supplemental Figure 1. Data selection process. [file 13048_2023_1095_MOESM1_ESM.pdf]
